# Peroxiredoxin activity is a major landmark of male fertility

**DOI:** 10.1038/s41598-017-17488-7

**Published:** 2017-12-07

**Authors:** Do-Yeal Ryu, Ki-Uk Kim, Woo-Sung Kwon, Md Saidur Rahman, Amena Khatun, Myung-Geol Pang

**Affiliations:** Department of Animal Science and Technology, Chung-Ang University, Anseong, Gyeonggi-do 456-756 Republic of Korea

## Abstract

Peroxiredoxins (PRDXs) are important antioxidant enzymes reported to have a role in sperm function and male fertility. However, how PRDXs affects male fertility remain fundamental unanswered questions. We therefore sought to investigate the role of these enzymes in sperm function and fertilisation. In this *in vitro* trial, mouse spermatozoa were incubated with different concentrations of conoidin A (1, 10, or 100 µM), a specific inhibitor of PRDXs. Our results demonstrated that inhibition of PRDXs by conoidin A significantly decreased the oxidized form of peroxiredoxins (PRDXs-SO_3_) in spermatozoa. Decreased PRDX activity was associated with a significant reduction in sperm motility parameters, viability, and intracellular ATP, whereas ROS levels, DNA fragmentation, and loss of mitochondrial membrane potential were increased. Simultaneously capacitation and the acrosome reaction were also significantly inhibited perhaps as a consequence of decreased tyrosine phosphorylation and protein kinase-A activity. In addition, fertilisation and early embryonic development were adversely affected following PRDXs inhibition in spermatozoa. Taken together, our data demonstrate that decreased PRDX activity directly affects male fertility due to negative effects on important functions and biochemical properties of spermatozoa, ultimately leading to poor fertilisation and embryonic development.

## Introduction

Mammalian spermatozoa must undergo several functional and physiological modifications prior to fertilisation. Among these, capacitation and the acrosome reaction are fundamental to sperm-oocyte fusion^1^. Both events are regulated by complex interactions that involve several signalling cascades, such as intracellular calcium influx, membrane fluidity, cyclic AMP (cAMP), protein kinase-A (PKA), and tyrosine phosphorylation^2–8^. Notably, reactive oxygen species (ROS) generation is one of the earliest events to occur during capacitation, and while this can induce toxicity, some levels can also be beneficial to facilitate capacitation in mammalian spermatozoa^9–12^.

It has been widely demonstrated that excessive ROS is potentially toxic to spermatozoa due to induction of DNA damage, oxidation of polyunsaturated fatty acids in lipids, amino acids, and proteins, and subsequent apoptosis^13^. One of the most vigorously researched ROS is H_2_O_2_, which affects sperm motility and causes DNA damage in spermatozoa by blocking oxidative metabolism^9,10,12^. However, there is evidence to suggest that spermatozoa actually require a small amount of H_2_O_2_ and other ROS molecules to maintain normal cellular function and fertility. De Lamirande *et al*. demonstrated that ROS, and specifically H_2_O_2,_ is primary factor in the induction of phosphorylation, through promotion of tyrosine phosphorylation, and thus controls capacitation in spermatozoa^11^. Therefore, in recent years there has been considerable interest in proteins that may regulate the maintenance of ROS.

Peroxiredoxins (PRDXs) are thought to be some of the major proteins that regulate ROS in almost all cell types. A recent study proposed that PRDXs are differentially expressed before and after capacitation in porcine spermatozoa^5^. In addition, Rahman *et al*. showed that PRDX5 is highly expressed in spermatozoa under stressed conditions to maintain cell survivability^14^. PRDXs are a well-conserved family of thiol-dependent peroxidases that regulate the antioxidant defence system in almost all cell types by reducing hydrogen peroxide (H_2_O_2_), peroxynitrite, and hydroperoxides. Abnormal function of PRDXs is associated with cancer, neoplasia, cardiovascular abnormalities, neurodegeneration, and male fertility^15–18^. In particular, an extensive literature search revealed that PRDXs play a critical role in the regulation of sperm function and male fertility^14,19–22^. Ozkosem *et al*. reported that a lack of PRDXs impairs sperm motility, capacitation, and DNA integrity^22^. Another recent study demonstrated that PRDXs levels are reduced in infertile male patients^23^. Thus, while there is a large body of knowledge suggesting a close relationship between PRDXs and male fertility, very little is known about the specific role that they play, and the underlying mechanism has not yet been elucidated. Therefore, the goal of our research is to evaluate the direct role of PRDXs in sperm function, fertility, and subsequently to clarify the molecular mechanism of PRDX function in spermatozoa using conoidin A, a specific PRDX inhibitor.

First, we measured PRDX activity in spermatozoa following conoidin A treatment using western blotting. Second, we examined the role of PRDXs in mouse sperm function, including motility, motion kinematics, viability, capacitation status, fertilisation, and early embryonic development. Finally, we analysed levels of intracellular ATP, mitochondrial membrane potential (MMP), ROS, DNA fragmentation index (DFI), lactate dehydrogenase (LDH; as a measure of cytotoxicity), PKA activity, and tyrosine phosphorylation in order to identify the molecular mechanism underlying PRDX inhibition in spermatozoa and male infertility.

## Results

### Conoidin A inhibits PRDX activity in spermatozoa

**Figure 1:**
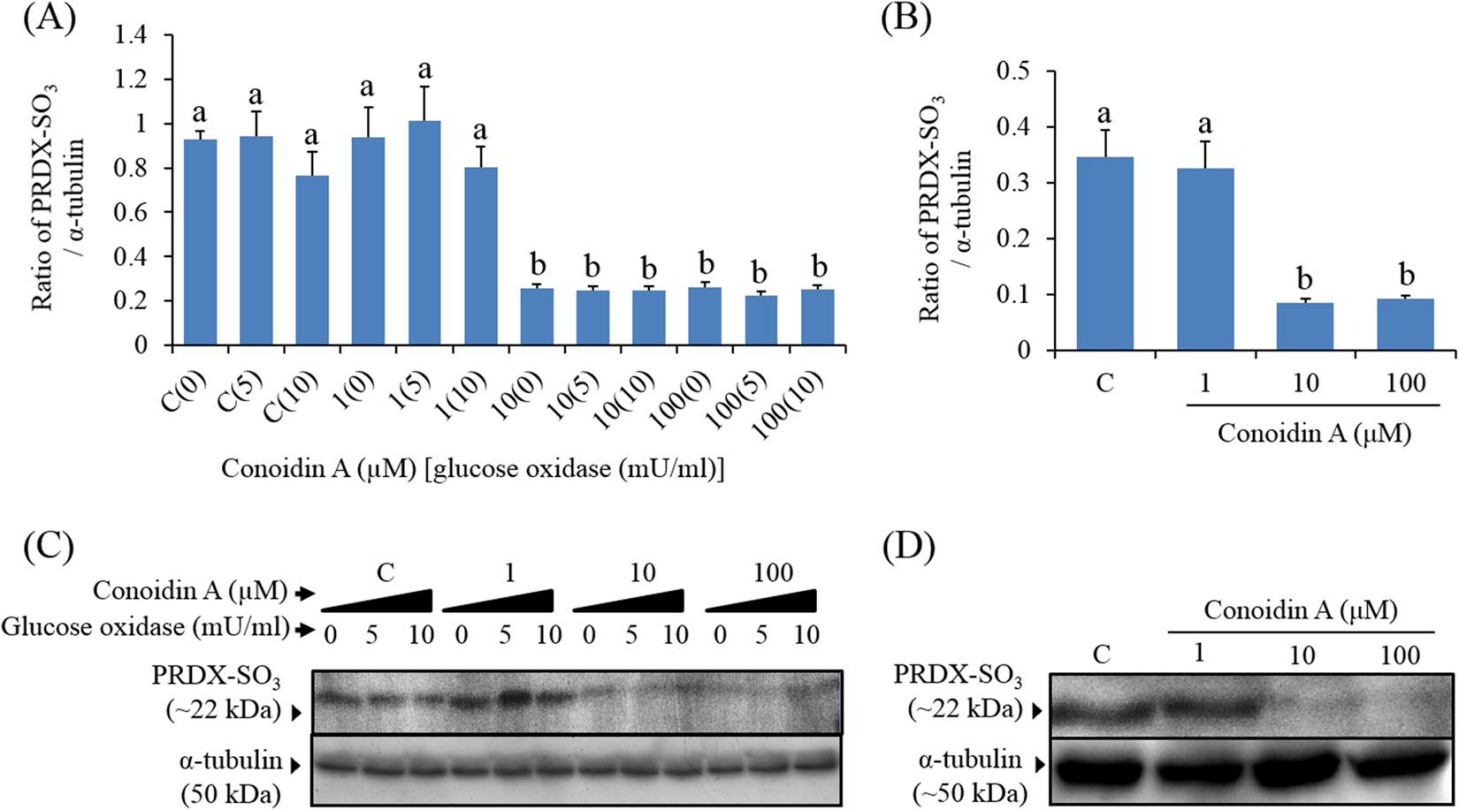
Conoidin A inhibits PRDX activity in mouse spermatozoa. (**A**) Density of PRDXs-SO_3_ in control and conoidin A-treated samples with exposure to glucose oxidase. (**B**) Density of PRDXs-SO_3_ in control and conoidin A-treated samples without exposure to glucose oxidase. (**C**) Representative western blot of PRDXs-SO_3_ probed with anti-peroxiredoxin-SO_3_ antibody (uncropped blots are presented in Supplementary Fig. 1). (**D**) Representative western blot of PRDXs-SO_3_ probed with anti-peroxiredoxin-SO_3_ antibody (uncropped blots are presented in Supplementary Fig. 1). Data represent mean ± SEM, n = 3. Values with superscripts (^a,b^) are significantly different between control and conoidin A-treatment groups by one-way ANOVA (*P* < 0.05) with Tukey’s multiple comparison test.

**Figure 2:**
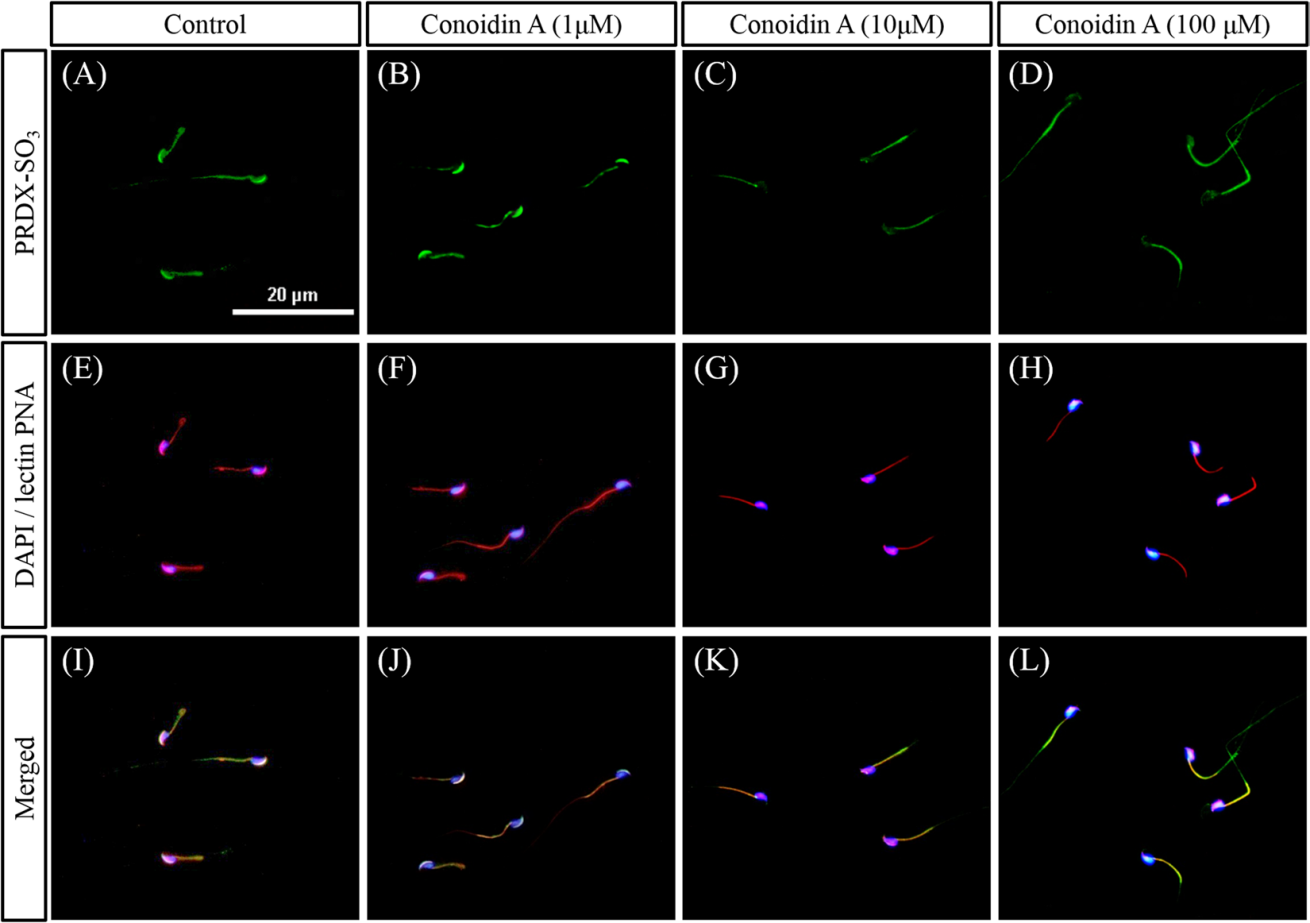
Expression levels of PRDXs-SO_3_ in spermatozoa following conoidin A treatment. Expression levels of PRDX-SO_3_ following exposure to different concentrations of conoidin A. (**A**–**D**) Images of PRDX-SO_3_ (green). (**E**–**H**) Merged images of nucleus (DAPI, blue) and acrosome (lectin PNA, red). (**I**–**L**) Merged images of nucleus, acrosome, and PRDX-SO_3._ Images were obtained using a Nikon TS-1000 microscope and NIS elements image software (Nikon, Japan). Bar = 20 μm.

To determine whether conoidin A inhibits PRDXs, we used western blot and immunofluorescence to measure levels of PRDXs-SO_3_ in spermatozoa. Our western blot results showed that conoidin A did indeed decrease PRDXs-SO_3_ levels in spermatozoa, with or without the addition of glucose oxidase (*P* < 0.001 and *P* = 0.001) (Fig. 1). In addition, our immunofluorescence results showed a decrease in PRDXs-SO_3_ expression in the acrosome of spermatozoa following treatment with higher concentrations (10 or 100 μM) of conoidin A (Fig. 2).

### Decreased PRDX activity reduces motility and motion kinematics of spermatozoa

CASA (computer-assisted sperm analysis) was used to evaluate the effect of PRDX inhibition on sperm motility and motion kinetics, and the results are shown in Table 1. The motility of the spermatozoa significantly decreased following treatment with higher concentrations of conoidin A (10 or 100 μM; Table 1, *P* < 0.001). Simultaneously, various kinematic parameters, such as hyperactivated motility (*P* = 0.039), curvilinear velocity (*P* = 0.002), straight-line velocity (*P* = 0.001), average path velocity (*P* < 0.001), amplitude of head lateral displacement (*P* = 0.001), linearity (*P* = 0.002), and wobble (WOB *P* = 0.002) were decreased at the highest concentration (100 μM) of conoidin A exposure (Table 1).

**Table 1:** Effect of decreased PRDX activity on sperm motility and motion kinematics.

Parameter	Control	Conoidin A		
		1 μM	10 μM	100 μM
MOT (%)	72.52 ± 1.22^a^	71.16 ± 1.33^a^	61.56 ± 0.35^b^	37.94 ± 1.71^c^
HYP (%)	20.52 ± 2.49^a^	17.63 ± 3.1^a,b^	19.43 ± 3.91^a,b^	6.97 ± 1.7^b^
VCL (μm/s)	149.03 ± 4.44^a^	145.82 ± 5.12^a^	149.33 ± 7.41^a^	110.66 ± 0.98^b^
VSL (μm/s)	56.99 ± 2.27^a^	57.79 ± 1.92^a^	59.95 ± 4.47^a^	33.31 ± 2.23^b^
VAP (μm/s)	66.56 ± 2.66^a^	67.07 ± 2.56^a^	67.85 ± 3.54^a^	43.71 ± 1.29^b^
ALH (μm)	6.27 ± 0.2^a^	5.99 ± 0.2^a^	6.12 ± 0.28^a^	4.54 ± 0.1^b^
LIN (%)	38.36 ± 0.76^a^	39.68 ± 1.12^a^	39.95 ± 0.96^a^	30.21 ± 1.92^b^
WOB (%)	44.65 ± 0.46^a^	46.04 ± 1.32^a^	45.36 ± 0.26^a^	39.62 ± 0.7^b^

Data presented as mean ± SEM, n = 3. MOT = motility (%), HYP = hyperactivated motility; VCL = curvilinear velocity (µm/s), VSL = straight-line velocity (µm/s), VAP = average path velocity (µm/s), ALH = mean amplitude of head lateral displacement (µm), LIN (%) = linearity, and WOB (%) = wobble. Superscripts (^a,b,c^) within the same row indicate significant differences (*P* < 0.05).

### Decreased PRDX activity reduces spermatozoa viability and intracellular ATP levels

**Figure 3:**
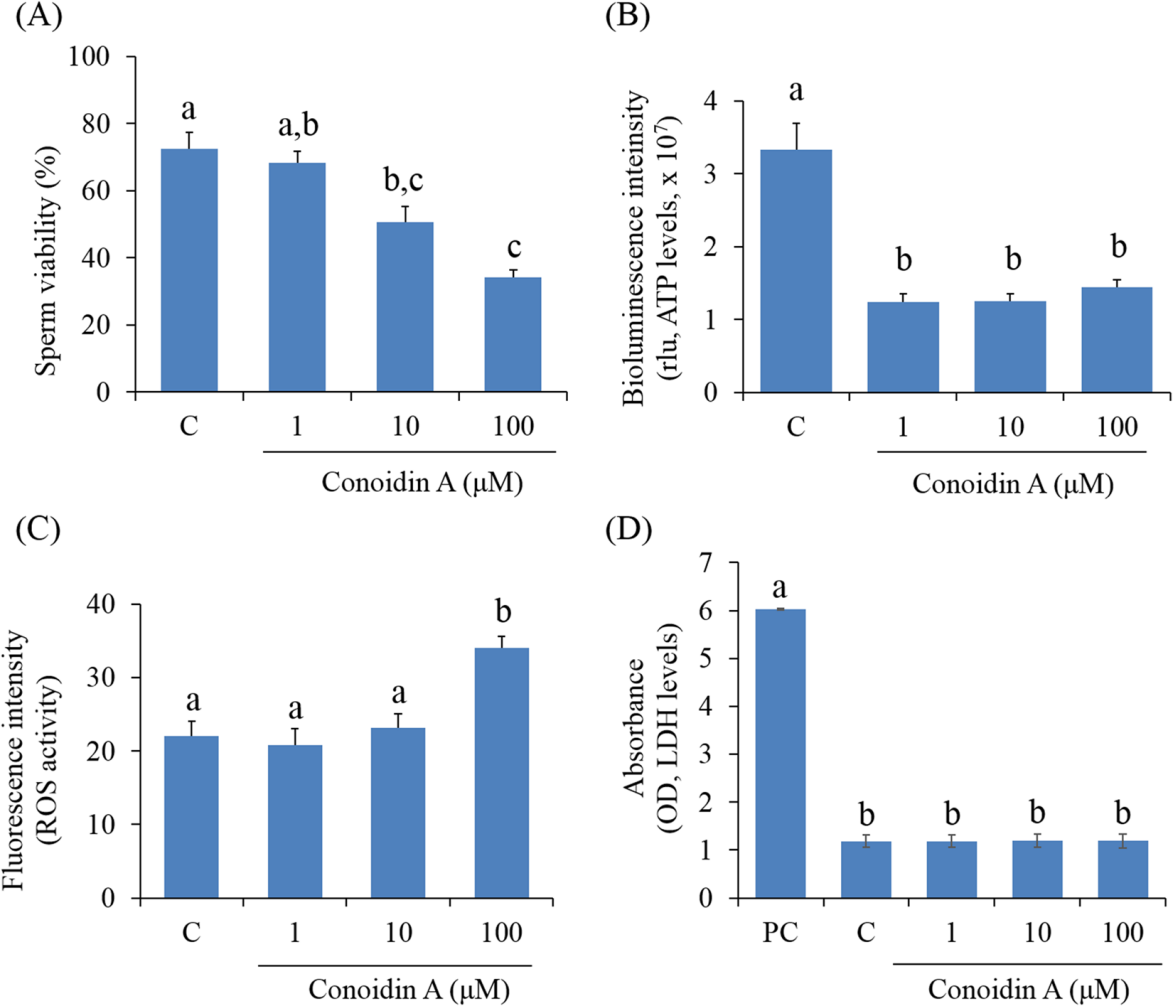
Effect of decreased PRDX activity on viability, intracellular ATP, reactive oxygen species (ROS), and lactate dehydrogenase (LDH) levels in mouse spermatozoa. (**A**) Changes in viability (%) of control and conoidin A-treated spermatozoa. (**B**) Differences in bioluminescence intensity (proportional to levels of intracellular ATP) between control and conoidin A-treated spermatozoa. (**C**) Differences in fluorescence intensity (proportional to intracellular ROS activity) between control and conoidin A-treated spermatozoa. (**D**) Differences in absorbance (proportional to levels of intracellular LDH) between positive control (PC), control and conoidin A-treated samples. Data are mean of three replicates ± SEM. Values with superscripts (^a,b,c^) are significantly different between control and treatment groups by one-way ANOVA (*P* < 0.05) with Tukey’s multiple comparison test.

Figure 3A shows a summary of the effects of PRDXs on sperm viability. According to the data, higher concentrations (10 or 100 μM) of conoidin A significantly decreased the number of viable spermatozoa compared to controls (*P* < 0.001). The fertilising capability of spermatozoa depends on a specific, time-dependent series of essential events, in which ATP plays a central role. Therefore, the effect of PRDXs on intracellular ATP levels were evaluated. ATP levels were found to be significantly lower in spermatozoa treated with 1, 10, or 100 μM conoidin A than in control spermatozoa (Fig. 3B, *P* < 0.001).

### Effect of decreased PRDX activity on intracellular ROS and LDH levels in spermatozoa

The effect of PRDXs on ROS and LDH levels in spermatozoa was evaluated. Intracellular levels of ROS were significantly higher in spermatozoa treated with 100 μM conoidin A than in control spermatozoa (Fig. 3C, *P* = 0.005). However, there was no significant difference in LDH levels between control and conoidin A-treated groups (Fig. 3D, *P* = 0.997).

### Decreased PRDX activity increases DFI and loss of MMP

**Figure 4:**
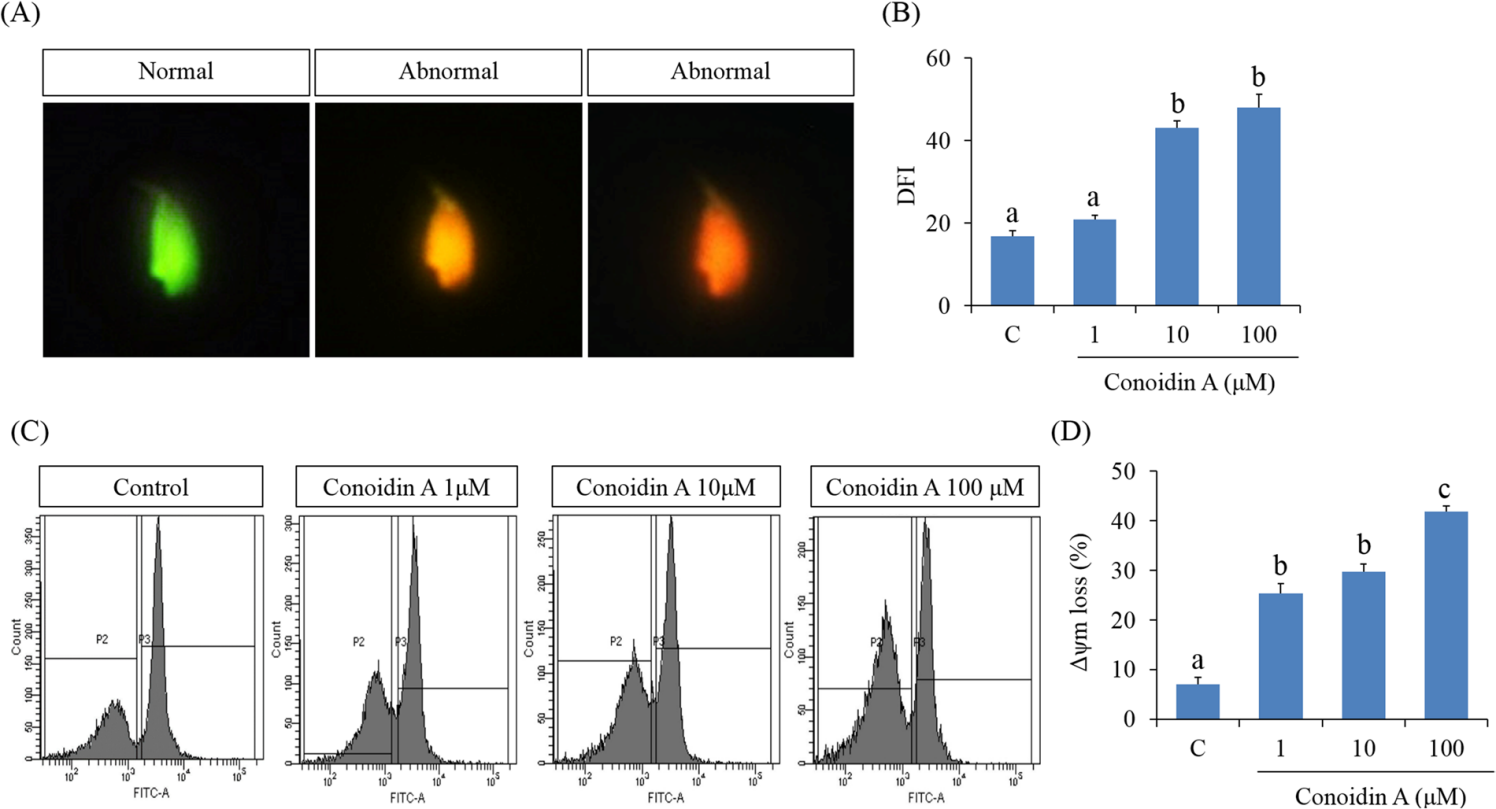
Effect of decreased PRDX activity on DNA fragmentation index (DFI) and mitochondrial membrane potential (MMP) in mouse spermatozoa. (**A**) Representative images of normal spermatozoa (green) and abnormal spermatozoa (orange and red) (**B**) Differences in fluorescence intensity (proportional to levels of DPI) between control and conoidin A-treated spermatozoa. (**C**) Representative flow cytometry images of MMP in control and conoidin A-treated spermatozoa (**D**) Differences in fluorescence intensity (proportional to levels of MMP loss) between control and conoidin A-treated samples. Data are mean of three replicates ± SEM. Values with superscripts (^a,b,c^) are significantly different between control and treatment groups by one-way ANOVA (*P* < 0.05) with Tukey’s multiple comparison test.

To determine the molecular mechanism(s) of inhibitory role of conoidin A, we also evaluated sperm DFI and MMP as shown in Fig. 4B,D, respectively. Our results revealed that high concentrations of conoidin A (10 or 100 μM) were able to significantly increased DFI in spermatozoa (Fig. 4B, *P* < 0.001). However, the loss of MMP increased significantly in all treatment groups compared to the control (Fig. 4D, *P* < 0.001).

### Effect of decreased PRDX activity on capacitation status of spermatozoa

**Figure 5:**
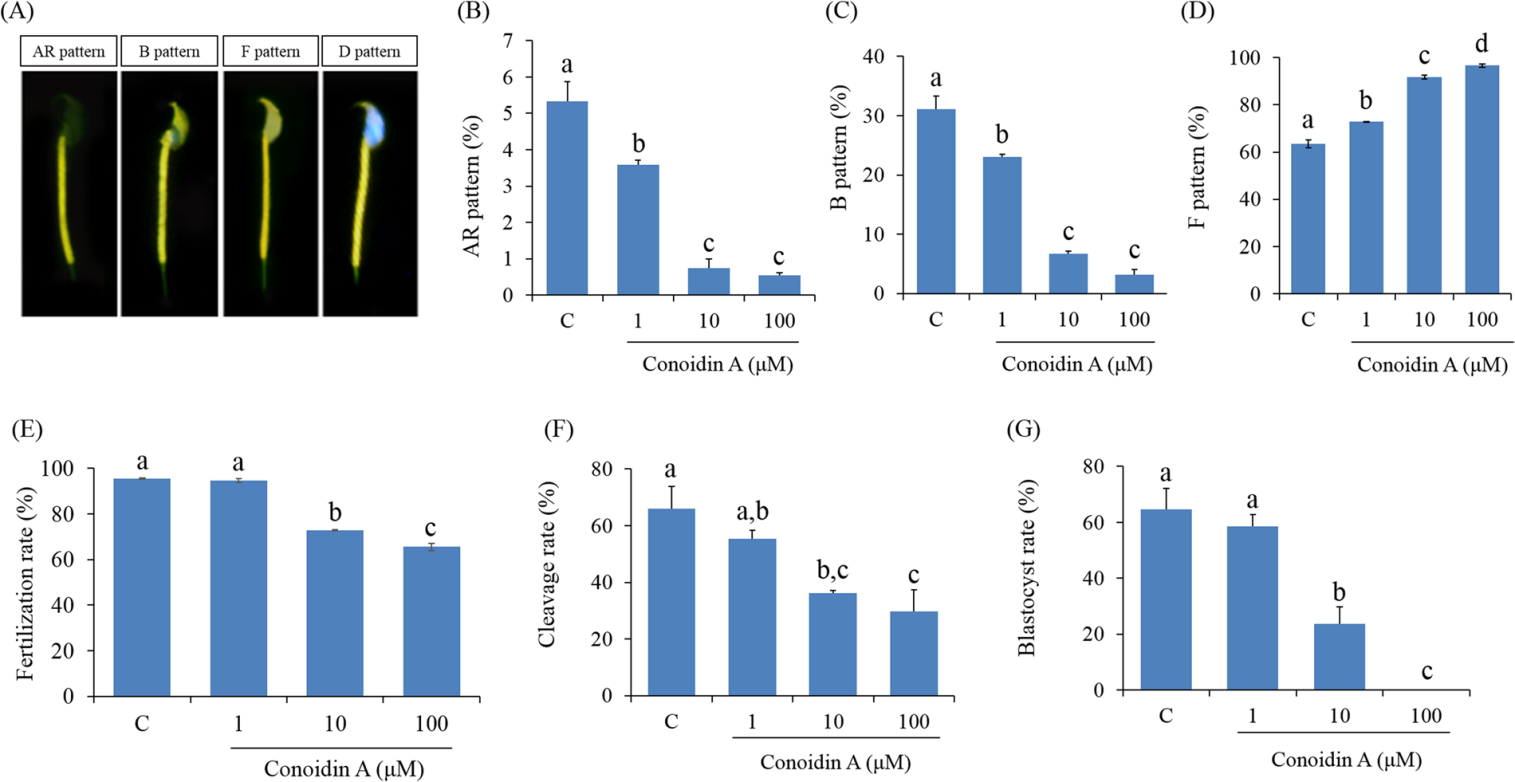
Effect of decreased PRDX activity on capacitation status, fertilisation, cleavage formation, and early embryonic development. (**A**) Representative image of acrosome-reacted (AR pattern), capacitated (B pattern), non-capacitated (F pattern), or dead (D pattern) spermatozoa. (**B**) Difference in percentage of AR spermatozoa under various treatment conditions after capacitation. (**C**) Difference in percentage of capacitated spermatozoa under various treatment conditions after capacitation. (**D**) Difference in the non-capacitated spermatozoa under various treatment conditions after capacitation. (**E**) Differences in fertilisation rate following IVF with spermatozoa treated with different conoidin A concentrations. (**F**) Differences in cleavage rate following IVF with spermatozoa treated with different conoidin A concentrations. (**G**) Difference in blastocyst formation rate following IVF with spermatozoa treated with different conoidin A concentrations. Data are mean ± SEM, n = 3. Values with superscripts (^a,b,c,d^) are significantly different between control and treatment groups by one-way ANOVA (*P* < 0.05) with Tukey’s multiple comparison test.

To evaluate the effect of PRDXs on the capacitation status of spermatozoa, a dual staining method was used. We found that the percentage of live capacitated and acrosome-reacted spermatozoa was significantly lower in conoidin A-treated spermatozoa than in control spermatozoa (Fig. 5B,C, *P* < 0.001). In addition, the number of live non-capacitated spermatozoa was significantly increased in the conoidin A-treated group, in a dose-dependent manner (Fig. 5D, *P* < 0.001).

### Effect of decreased PRDX activity on fertilisation capacity of spermatozoa and subsequent embryonic development

We used *in-vitro* fertilisation (IVF) system to evaluate the effect of PRDXs in spermatozoa on fertilisation and embryonic development. The fertilisation and cleavage rate using conoidin A-treated (10 and 100) spermatozoa were significantly lower than for IVF using control spermatozoa (Fig. 5E and F, *P* < 0.001*, P* = 0.006). The rate of blastocyst formation was also significantly decreased using spermatozoa treated with higher doses of conoidin A compared to controls (Fig. 5G, *P* < 0.001).

### Regulatory mechanism underlying PRDX inhibition in spermatozoa

**Figure 6:**
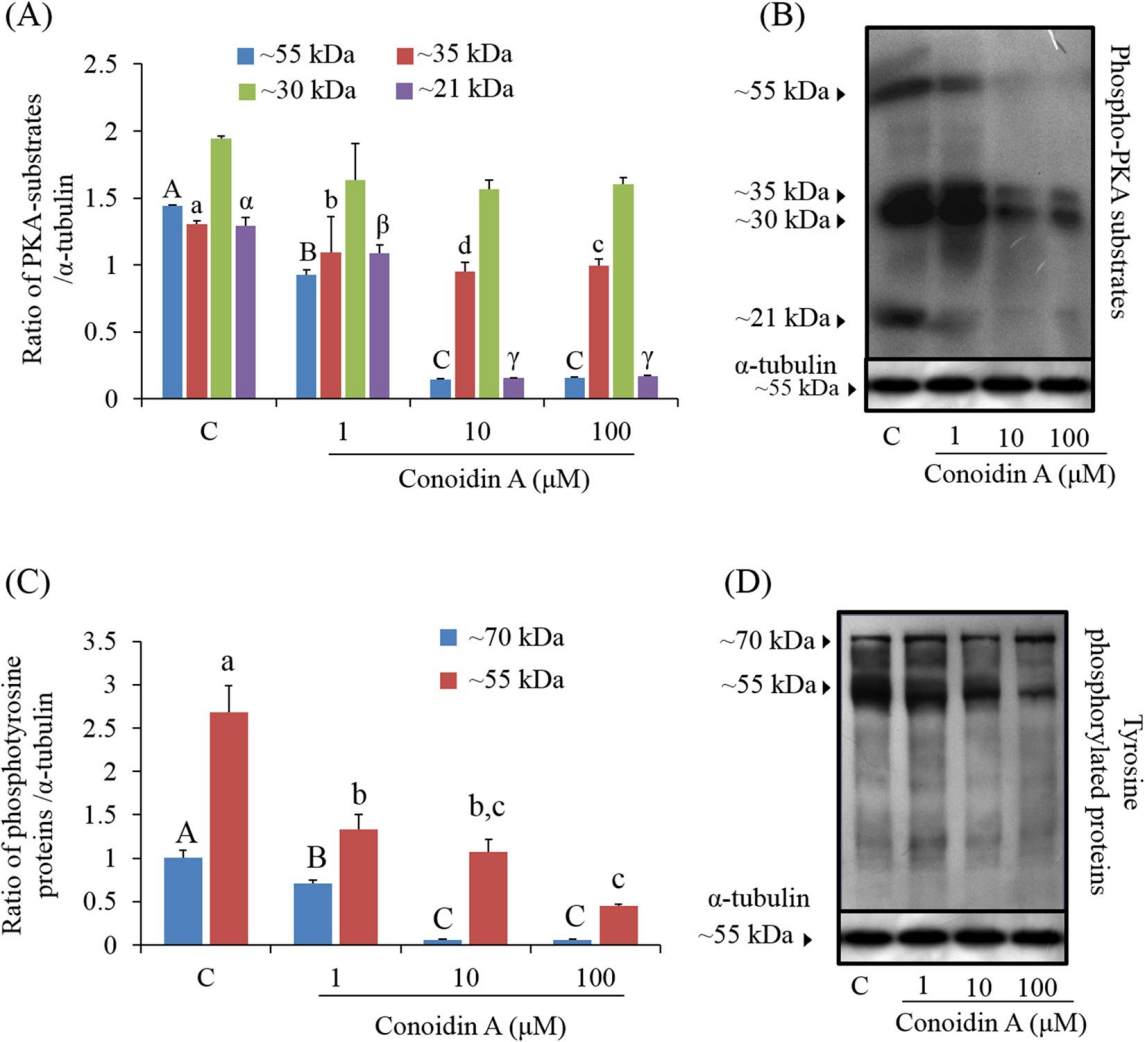
Effect of decreased PRDX activity on PKA activity and tyrosine phosphorylation. (**A**) Density of PKA substrates in control and conoidin A-treated samples (blue bar: ~55 kDa, red bar: ~35 kDa, green bar: ~30 kDa, purple bar: ~21 kDa). (**B**) Phospho-PKA substrates were probed with an anti-phospho-PKA antibody; lane 1: Control, lane 2: 1 µM conoidin A, lane 3: 10 µM conoidin A, lane 4: 100 μM conoidin A (uncropped blots are presented in Supplementary Fig. 1). (**C**) Density of tyrosine-phosphorylated proteins in control and conoidin A-treated spermatozoa (blue bar: ~70 kDa, red bar: ~55 kDa). (**D**) Tyrosine-phosphorylated proteins were probed with an anti-phosphotyrosine antibody; lane 1: Control, lane 2: 1 µM conoidin A, lane 3: 10 µM conoidin A, lane 4: 100 μM conoidin A (uncropped blots are presented in Supplementary Fig. 1). Data represent mean ± SEM, n = 3. Values with superscripts (^A, B, C, a, b, c, d, α, β, γ^) are significantly different between control and conoidin A-treatment groups by one-way ANOVA (*P* < 0.05) with Tukey’s multiple comparison test.

To assess the regulatory mechanism underlying the effect of PRDX inhibition in spermatozoa, PKA activity and phosphotyrosine levels were measured in conoidin A-treated and untreated control spermatozoa (Fig. 6). Levels of four different PKA substrate species (~21, ~30, ~35, and ~55 kDa) were lower in treated spermatozoa that in control spermatozoa, with a particularly noticeable decrease in the ~21, ~35, and ~55 kDa species (Fig. 6A,B, *P* < 0.001). Likewise, tyrosine-phosphorylated species (~55 and ~70 kDa) were also significantly decreased in conoidin A-treated spermatozoa compared to controls (Fig. 6C,D, *P* < 0.001).

### Bioinformatics information of PRDXs

**Figure 7:**
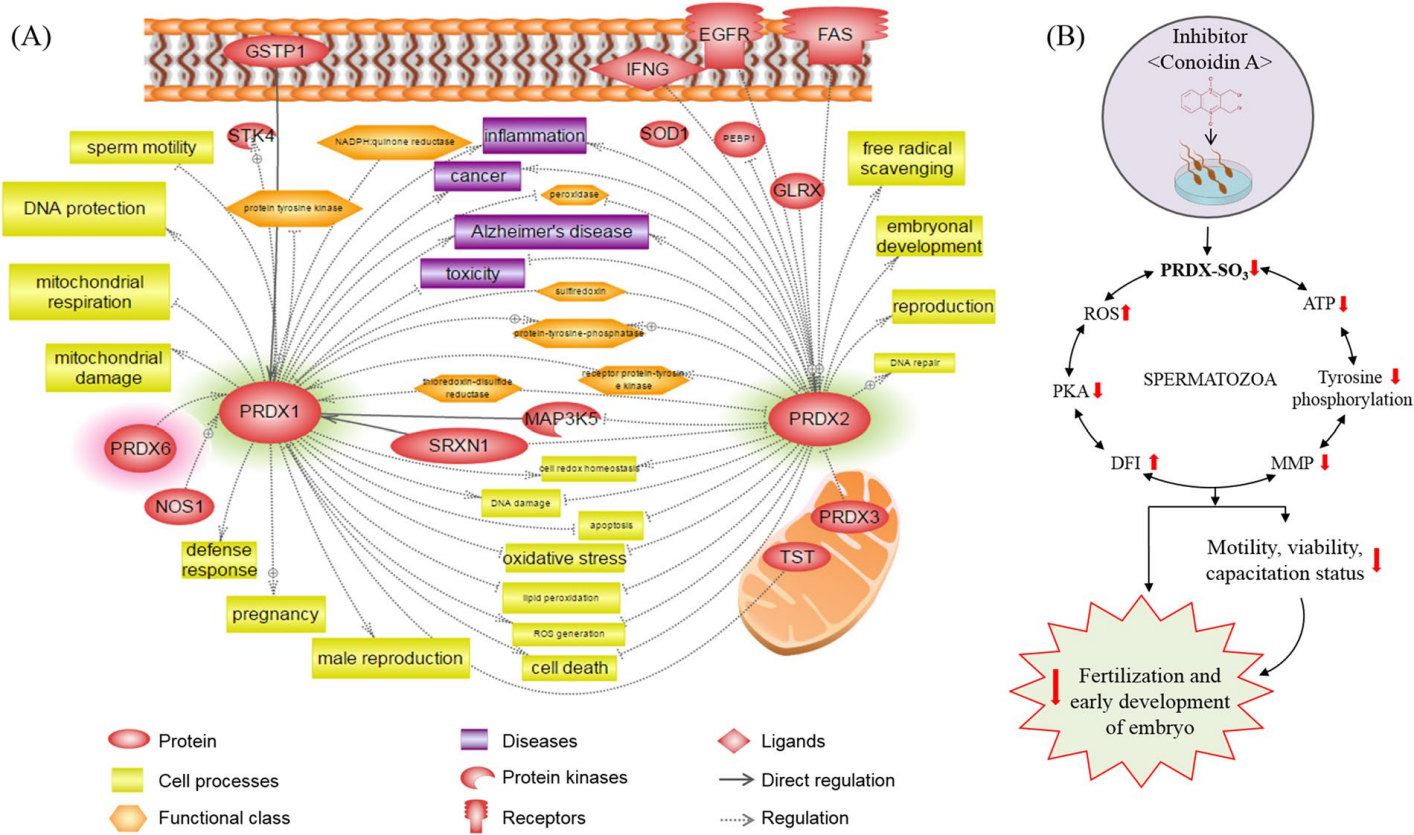
Signalling pathways associated with PRDXs and hypothetical illustration of PRDXs in mouse spermatozoa. (**A**) Protein-protein interactions, cellular regulations, and associated diseases related to PRDX activity illustrated using the Pathway Studio program. (**B**) Hypothetical illustration demonstrates the effect of conoidin A on sperm function and fertilisation. Conoidin A decreases PRDX activity in spermatozoa, which is associated with the alteration of several sperm functions, including motility, viability, and capacitation status, perhaps as a consequence of altered reactive oxygen species (ROS), DNA fragmentation index (DFI), mitochondrial membrane potential (MMP), ATP levels, PKA activity, and tyrosine phosphorylation. All of these changes ultimately affect fertilisation and early embryonic development.

To investigate the functional role of PRDXs with interacting proteins, cellular regulation, and associated diseases, which can affect sperm function and fertility, Pathway Studio was used as a bioinformatics tool. We found that PRDXs interact with several proteins, including glutathione S-transferases, nitric oxide synthase 1, serine/threonine kinase 4, PRDX 3 and 6, mitogen-activated protein kinase 5, superoxide dismutase1, and Fas cell surface death receptor. In addition, we showed that these proteins affect several important cellular processes such as free radical scavenging, DNA repair, defence response, mitochondrial damage, sperm motility, and ultimately male reproduction. Finally, we showed that these functional roles of PRDXs are closely related to several diseases, such as inflammation, cancer, Alzheimer’s disease, and toxicity (Fig. 7).

## Discussion

PRDXs are antioxidant enzymes, which are present in almost all cell types and function to maintain cell homeostasis by regulating H_2_O_2_ levels. Since H_2_O_2_ was discovered as a primary factor to induce capacitation, PRDXs are increasingly recognized as a vital factor in male fertility. There are ample information that oxidative stress may be involved in male infertility. Therefore it is tempting to speculate that PRDX can be important factors in male infertility. Several researchers have demonstrated that lower levels of PRDXs lead to an inability to maintain intracellular ROS levels, causing oxidative stress, apoptosis in spermatozoa, and ultimately leading to male infertility. However, the direct role of PRDXs in sperm function and fertility has not yet been elucidated. In the current study, we investigated the role and underlying mechanism of PRDXs in sperm function and fertility using an antagonistic approach in an *in vitro* system.

Previous studies that investigated the role of PRDXs in human epithelial cells, used conoidin A with an additional H_2_O_2_ donor, glucose oxidase^24^. Conoidin A is a cell-permeable crystalline solid compound that has been reported to inhibit PRDX activity, thus blocking oxidation to sulfinic acid (SO_2_H) and sulfonic acid (SO_3_) by covalently binding to catalytic cysteines^24,25^. In the current study, we measured levels of PRDXs-SO_2_H/SO_3,_ an oxidized form of PRDXs, to determine whether PRDXs in spermatozoa are inhibited by conoidin A treatment. Indeed, there was a significant reduction in the levels of PRDXs-SO_3_ in conoidin A-treated (10 and 100 μM) groups compare with untreated spermatozoa, under both elevated and non-elevated H_2_O_2_ conditions. Unexpectedly, there was no significant change in PRDXs-SO_3_ levels in spermatozoa from the glucose oxidase-treated groups. It is known that spermatozoa continuously generate low amounts of ROS for capacitation and hyperactivation^26^. Therefore, these results suggest that additional H_2_O_2_ does not affect the levels of PRDXs-SO_3_ in spermatozoa, unlike that reported for human epithelial cells^24^.

It is well known that motility and morphology are the most important characteristics for the fertilising capability of mammalian spermatozoa^5,27^. Therefore, they are frequently measured in clinics and research laboratories to evaluate the fertilising competency of spermatozoa. In the present study, we evaluated the effect of decreased PRDX levels on motility/motion kinematics and viability of mouse spermatozoa using conoidin A treatment. Both motility and viability were significantly affected as a result of PRDXs inhibition by higher doses of conoidin A (10 or 100 μM). Simultaneously, motion kinematics parameters such as HYP, VCL, VSL, VAP, ALH, LIN, and WOB were affected by inhibition of PRDX activity using highest concentration of conoidin A. It has been reported that reduced ATP levels and increased oxidative stress are the primary reasons for loss of sperm motility and motion kinematics^14,28,29^. Mukai and Okuno demonstrated that mitochondrial respiration generates ATP, which supports sperm motility and motion kinematics^30^. Furthermore, there is ample experimental evidence showing that low levels of antioxidant enzymes in spermatozoa are closely related to oxidative stress. Mammalian spermatozoa are highly susceptible to ROS, which can damage membranes through lipid peroxidation^31^. Moreover, elevated ROS leads to sperm plasma membrane disruption, DNA damage, mitochondrial dysregulation, and prohibition of capacitation in spermatozoa^32,33^. Impaired mitochondrial function also affects ATP generation^32^. Therefore, to better explain the loss of sperm motility and motion kinematics, we measured intracellular ROS, ATP, and MMP (Δψm) in spermatozoa. Our results demonstrated that all doses of conoidin A were decreased intracellular ATP levels and MMP, whereas ROS levels were increased at highest concentration. Therefore, all these changes could affect sperm motility and motion kinematics. It has been demonstrated that damages to the sperm DNA and its substrates are negatively correlated with sperm motility, motion kinematics, and viability^34–37^. As we noticed decreased PRDX activity in a conoidin A-containing microenvironment was associated with increased DFI in spermatozoa, thus could affect sperm motility, motion kinematics and viability.

Recent studies demonstrated that extreme levels of ROS increase LDH levels, which is associated with apoptosis, and damages the integrity of sperm DNA^38^. Unexpectedly, in our study, although ROS levels and DFI were increased following PRDX inhibition, there was no significant difference in LDH levels between treated and untreated spermatozoa. Therefore, inhibition of PRDXs affects motility/motion kinematics and sperm viability, but does not appear to be toxic to spermatozoa. It is also important to note that although 1 μM of conoidin A did not inhibit PRDXs activity, it was capable to alter several sperm parameters (e.g., intracellular levels of ATP, LDH, MMP, and capacitation status). Therefore, further studies are required to investigate low dose effect of conodin A (~1 μM) on spermatozoa and their correspondence relationship with PRDXs activity.

Capacitation and the acrosome reaction are required for successful fertilisation both *in vivo* and *in vitro* ^14,39–41^. During capacitation, spermatozoa gain hyperactive motility and reach the zona pellucida. Then, these capacitated spermatozoa undergo the acrosome reaction and are able to fuse with the oocyte plasma membrane^1^. As these events progress, intracellular levels of cAMP, PKA activity, and protein tyrosine phosphorylation are increased^3,42^. In the current study, inhibition of PRDXs significantly affected the ability of spermatozoa to become capacitated and undergo the acrosome reaction. Simultaneously, similar effects on capacitation and acrosome reaction were also noticed while treated with 1 μM of conoidin A. To elucidate the underlying molecular mechanism, we evaluated PKA activity and protein tyrosine phosphorylation levels, and showed that both were significantly decreased in spermatozoa following treatment with conoidin A. Recent research also demonstrated that lower levels of PKA substrates and tyrosine phosphorylation were found in treated spermatozoa compared to control in humans^21^. Consistent with previous findings, it is plausible to suggest that PRDXs may regulate protein tyrosine phosphorylation in spermatozoa via a PKA-dependent mechanism. Additionally, Turner *et al*. reported that cAMP-dependent and PKA-mediated phosphorylation of flagellar proteins regulate sperm motility^43^.

Another finding of the current study is that spermatozoa with inhibited PRDXs displayed a significant decrease in fertilisation, cleavage, and embryonic development rates compared to the control group, in an *in vitro* fertilisation system. It has been reported that spermatozoa with higher DNA damage are responsible for fertilisation failure and compromised embryo development^44^. Therefore, decreased fertilisation, cleavage, and early embryo development noticed in current study might be contributed via increased DNA damages (DFI) in spermatozoa. Likewise, Ozkosem *et al*. reported that an absence of PRDX6 resulted in a decreased litter size in the treated group compared to the controls^22^. In addition, the decreased number of capacitated and the acrosome-reacted spermatozoa noticed in current study may also affect the fertilisation competence.

In addition, we used Pathway Studio to investigate PRDXs-interacting/associated proteins and the regulatory mechanism of PRDXs in cellular functions and diseases. Consistent with our experimental findings, Pathway Studio identified that PRDX 1 and 2 can regulate ROS generation, free radical scavenging, oxidative stress, lipid peroxidation, mitochondrial damage, sperm motility, apoptosis, and male fertility. In addition, we found that PRDXs interact with several proteins that are related to sperm function, such as serine/threonine kinase 4, mitogen-activated protein kinase 5, superoxide dismutase 1, glutathione S-transferase 1, sulfiredoxin 1, thiosulfate sulfurtransferase, and glutaredoxin.

Recent insightful evidence has improved the prognosis and diagnosis of male fertility via the discovery of biomarkers. Among them are critical proteins that represent potential biomarkers for diagnosing male fertility, such as dehydrogenase, PRDX 4 and 5, phospholipid hydroperoxide glutathione peroxidase, ubiquinol-cytochrome-c reductase complex core protein 2, and glutathione S-transferase Mu 5^45-47^. Intriguingly, these proteins are widely known to be important antioxidant enzymes that regulate sperm function and male fertility. Based on our bioinformatics data and previous studies, we summarize that a decrease in PRDX activity affects sperm function and fertility via several complex interactions, in particular the antioxidant system in spermatozoa.

As illustrated in Fig. 7, our data demonstrate that inhibition of PRDXs in spermatozoa directly affects male fertility due to negative effects on important functional and biochemical properties. Thus, current findings provide a strong platform from which to design new methods for effective male contraception through targeting PRDXs, which would block male fertility without any cytotoxic effects. However, further studies are needed to evaluate the safety of conoidin A as a contraceptive agent. In addition, PRDXs may be a novel candidate biomarker for diagnosing male fertility in humans and other mammals.

## Methods

### Ethics statement

All procedures were approved by the Institutional Animal Care and Use Committee (IACUC) of Chung-Ang University, Seoul, Republic of Korea. Experiments were performed according to the IACUC guidelines for the ethical treatment of animals.

### Media and chemicals

Unless otherwise specified, all chemicals were purchased from Sigma-Aldrich (St. Louis, MO, USA). Modified Tyrode’s medium was used as the basic medium (BM)^38,41^. Culture medium was pre-incubated and 0.4% bovine serum albumin (BSA) was added to induce capacitation the day before experiments were started. Conoidin A, a specific PRDX inhibitor, (Cayman Chemical Company, Ann Arbor, MI, USA) was diluted in dimethyl sulphoxide (DMSO) and stored at −20 °C. This stock solution was diluted in BM to obtain working concentrations of 1, 10, and 100 mM. Glucose oxidase was diluted in 50 mM sodium acetate buffer (pH 5.1) and stored at 0 °C. This stock solution was diluted in BM to obtain working concentrations of 5 and 10 mU for western blot sample preparation.

### Sperm preparation and treatment

The cauda epididymis of 8–12-week-old male ICR mice (Nara Biotech, Seoul, Korea) was isolated and spermatozoa were collected according to standard procedures^39,41^. Briefly, both cauda epididymides were separated from the surrounding fat and tissue, and placed in a sterile culture dish containing 2 mL BM with 0.4% BSA. Sperm suspensions were released with a sterile disposable syringe then incubated at 37 °C for 12 min in an incubator with 5% CO_2_ to disperse the spermatozoa^48^. Next, the dispersed spermatozoa were incubated for 90 min with various concentrations of conoidin A (1, 10, and 100 mM) to induce capacitation.

### Western blot analysis of phospho-PKA substrates, tyrosine phosphorylation, and oxidized PRDXs

Western blot analysis of PKA activity, tyrosine phosphorylation, and oxidized PRDXs (as PRDX-SO_3_) in mouse spermatozoa was performed as previously described^24,48,49^. Briefly, after capacitation each sample was washed three times with DPBS and centrifuged at 10,000 × *g* for 10 min. The supernatant was removed, and sperm pellets were resuspended in Laemmli sample buffer (63 mm Tris, 10% glycerol, 10% sodium dodecyl sulphate, 5% bromophenol blue) containing 5% 2-mercaptoethanol and incubated at room temperature for 10 min. After incubation, the treated samples were centrifuged at 10,000 × *g* for 10 min, and cell pellets were boiled at 100 °C for 3 min. Samples were resolved by SDS-PAGE using a 12% mini-gel system (Amersham, Piscataway, NJ, USA), and the separated proteins were transferred to a polyvinylidene fluoride membrane (Amersham). The membrane was blocked for 1 h at room temperature with blocking agent (3%; Amersham). To detect phosphor-PKA substrates, the membrane was incubated overnight with a rabbit monoclonal anti-phospho-PKA antibody (1:10,000; Cell Signaling Technology, Danvers, MA, USA) at 4 °C, then with a horseradish peroxidase (HRP)-conjugated goat anti-rabbit IgG (Abcam) diluted 1:5000 for 1 h at room temperature. Tyrosine phosphorylation was detected using an HRP-conjugated mouse monoclonal anti-phosphotyrosine antibody (PY20, 1:2500; Abcam) overnight at 4 °C. PRDXs-SO_3_ was detected using a rabbit polyclonal anti-PRDXs-SO_3_ antibody (1:2000; Abcam) after treating the sample with/without glucose oxidase. α-tubulin was used as an internal control (detected with a mouse monoclonal anti-α-tubulin antibody, 1:10000; Abcam) for 2 h at room temperature. The membrane was washed three times with PBS-T, and protein-antibody complexes were visualized using enhanced chemiluminescence. Bands were scanned using a GS-800-calibrated imaging densitometer (Bio-Rad, Hercules, CA, USA) and analysed using Quantity One software (Bio-Rad). Finally, the ratios of phospho-PKA substrate/α-tubulin, phosphotyrosine/α-tubulin, and Prxs-SO_3_/α-tubulin were calculated. Three male mice per replicate were used for each independent western blot experiment.

### Immunofluorescence

Immunofluorescence was conducted using an anti-PRDXs-SO_3_ antibody to visualize PRDXs-SO_3_ expression in control and treated samples. Briefly, mouse spermatozoa were air-dried and fixed with 3.7% paraformaldehyde for 30 min at 4 °C^50^. Air-dried slides were washed with Dulbecco’s phosphate-buffered saline (DPBS) containing 0.1% Tween 20 (PBS-T) and blocked for 1 h in blocking solution (5% BSA in PBS-T) at 4 °C. After blocking, slides were incubated with rabbit polyclonal primary antibodies against peroxiredoxins-SO_3_ (Abcam) (1:100 in blocking solution), or lectin PNA 34 and 35 conjugated to Alexa Fluor 647 (Molecular Probes) (1:100 in blocking solution) overnight at 4 °C. Next, slides were washed with PBS-T and incubated at room temperature for 2 h with a fluorescein isothiocyanate-conjugated goat polyclonal rabbit IgG secondary antibody (Abcam) (1:100 in blocking solution). Finally, slides were counterstained with Hoechst 33342, mounted with antifade reagent and imaged using a Nikon TS-1000 microscope and NIS Elements image software (Nikon, Tokyo, Japan).

### Detection of sperm motility by CASA system

Sperm motility and kinematic parameters were measured using the CASA system (SAIS Plus version 10.1; Medical supply, Seoul, Korea)^51^. Briefly, 10 μL of the sperm suspension was placed in a Makler chamber (Makler, Haifa, Israel), which was placed on the heated plate (37 °C) of a microscope. Spermatozoa were detected using a 10× phase contrast objective, and SAIS software was used for analysis. The program setting was specified previously (frames acquired, 20; frame rate, 30 Hz; minimum contrast, 7; minimum size, 5; low/high size gates, 0.4–1.5; low/high intensity gates, 0.4–1.5; non-motile head size, 16; non-motile brightness, 14). Hyper-activated (HYP) spermatozoa were measured as curvilinear velocity (VCL) ≥150 µm/sec, mean amplitude of head lateral displacement (ALH) ≥5 µm/sec, and linearity (LIN) ≤50% as reported previously^39,52,53^.

### Hypo-osmotic swelling test (HOST)

To evaluate sperm viability and membrane integrity, we used the hypo-osmotic swelling test (HOST) as previously described^54^. Briefly, 100 µL of sperm suspension from each treatment and control group was added to 900 µL hypo-osmotic solution (distilled water: 0.9% NaCl [1:1], 150 mOsm/kg) and incubated at 37 °C for 30 min. After incubation, 10 μL of each sample was placed onto a slide, spread using a coverglass, and allowed to air-dry. Spermatozoa were examined using a Microphot-FXA microscope (Nikon, Osaka, Japan) with a 20× objective (Nikon, Osaka, Japan). Sperm swelling patterns were observed using a Microphot-FXA microscope and classified broadly as viable or nonviable according to WHO 2010 guidelines.

### Measurement of intracellular ATP

Intracellular ATP concentration was measured using an ATP Bioluminescence Assay Kit HS II (Roche Molecular Biochemicals, Mannheim, Germany), according to the manufacturer’s instructions and previous studies^48,51^. Briefly, spermatozoa were diluted to 10^5^–10^8^ cells/μL, of which 25 μL was plated onto a 96-well plate. Spermatozoa were treated with an equal volume of cell lysis reagent and incubated at room temperature for 5 min. Luciferase reagent (50 μL) was added to each well immediately before measurement. ATP bioluminescence intensity (RLU) was measured using a Microplate Multimode Reader (GloMax-Multi Microplate Multimode Reader; Promega, Madison, WI, USA). As such, the RLU value provides a qualitative measurement of ATP levels in the cells.

### Measurement of intracellular ROS

Qualitative detection of cellular ROS was performed using the oxidation-sensitive fluorescent dye DCFDA (Abcam, Cambridge, UK) as previously described^38,55^. Fluorescence of treated and control samples was detected with a microplate fluorometer (Gemini Em; Molecular Devices, Sunnyvale, CA, USA) and analysed using SoftMax Pro 5 (Molecular Devices). As such, fluorescence intensity is proportional to the level of intracellular ROS.

### Measurement of intracellular LDH

To measure cytotoxicity, we used calorimetric detection of released LDH from spermatozoa using a CytoTox 96 Nonradioactive Cytotoxicity assay kit (Promega). Briefly, each sample was counted, lysis buffer was added, and samples were incubated at 37 °C in 5% CO_2_ for 1 h. After incubation, samples were centrifuged at 250 × *g* for 4 min. LDH positive control solution (2 µl of LDH positive control in 10 ml of PBS + 1% BSA) was prepared according to manufacturer guideline. The supernatant was transferred to a 96-well plate containing substrate (50 μL/well) and incubated for an additional 30 min at room temperature in the dark. After adding 50 μL stop solution, absorbance of each sample was measured, and LDH activity was computed as absorbance (OD) using SoftMax Pro 5 software.

### Detection of sperm DFI

Acridine orange staining was performed to determine the sperm DNA integrity. Briefly, mouse spermatozoa were air-dried and fixed overnight in Carnoy’s solution (methanol/acetic acid, 3:1). After fixing, slides were air-dried and washed with distilled water. Next, slides were stained with acridine orange solution for 5 min. Acridine orange solution (10 ml of 1% AO in distilled water added to mixture of 40 ml of 0.1 M citric acid and 2.5 ml of 0.2 M Na_2_HPO_4_7H_2_O) was prepared and stored in dark at 4 °C. After proper washing and drying, slides were examined using a Microphot-FXA microscope with a 460-nm filter immediately. This analysis indicated three types of sperm DNA integrity: normal DNA integrity (green fluorescence), abnormal DNA integrity (orange/red fluorescence). The DFI, which is the ratio of the orange/red (abnormal DNA integrity) to the total (orange/red + green) was calculated for the samples. At least 200 spermatozoa were evaluated per slide for each treatment^56–58^. Representative images of normal spermatozoa (green) and abnormal spermatozoa (orange and red) are shown in Fig. 4A.

### Measurement of sperm MMP

Mitochondrial integrity was measured by rhodhamine 123 as described previously^59^. Briefly, spermatozoa was washed with PBS and adjusted to 5 × 10^6^/mL concentration. Next, rhodhamine 123 (Rh123) was added to spermatozoa and incubated for 20 min at the room temperature in the dark. After incubation, spermatozoa were centrifuged at 100 g for 3 min. The sperm pellets were added to 1 mL of PBS and analysed with a flow cytometer. Flow cytometric analyses were performed using the Dual-Laser FACS Aria II (BD Biosciences, San Jose, CA, USA). The fluorescence intensities of Rh123 and PI of 10,000 events were recorded for each treatment. Spermatozoa with normal mitochondrial integrity, which is expressed by positive signal for Rh123 and negative signal for PI was calculated^59,60^.

### Combined H33258/chlortetracycline fluorescence (H33258/CTC) assessment of spermatozoa

CTC staining assays were performed to determine the capacitation status of spermatozoa using a dual-staining method^48,49^. Briefly, 15 μL H33258 solution was added to 135 μL of each sample and incubated for 2 min at room temperature. Then, 250 μL 2% polyvinylpyrrolidone in Dulbecco’s phosphate-buffered saline (DPBS) was added, and samples were centrifuged at 100 × *g* for 2.5 min to remove excess dye. After centrifugation, the supernatant was removed, the cell pellet was resuspended in 100 μL DPBS, and 100 μL CTC solution was added. Capacitation status was observed using a Microphot-FXA microscope with ultraviolet BP 340–380/LP 425 and BP 450–490/LP 515 excitation/emission filters for H33258 and CTC, respectively. This analysis indicated four patterns of capacitation status: dead (D pattern, blue fluorescence), non-capacitated (F pattern, bright yellow fluorescence distributed uniformly over the entire sperm head), capacitated (B pattern, bright yellow fluorescence over the acrosomal region and a dark post-acrosomal region), or acrosome-reacted (AR pattern, no fluorescence over the head, or yellow fluorescence only in the post-acrosomal region) as previously reported^61^. At least 400 spermatozoa were evaluated per slide for each treatment.

### ***In vitro*** fertilisation (IVF)

Eight- to twelve-week-old female hybrid B6D2F1/CrljOri mice (Nara Biotech, Seoul, Korea) were used for IVF. Mice were superovulated with an intraperitoneal injection of 5 IU pregnant mare serum gonadotropin (PMSG) followed 2 days later by 5 IU human chorionic gonadotropin (hCG)^49^. Fifteen hours after hCG injection, cumulus-oocyte complexes (COCS) were gathered from the ampulla in DPBS in a sterile cell culture dish. The COCs were placed in 50 μL BM containing 10% foetal bovine serum (FBS) under mineral oil and incubated at 37 °C in 5% CO_2_ for 1 h before insemination. To induce sperm capacitation, conoidin A-treated spermatozoa were washed with BM supplemented with 0.4% BSA. The COCs were inseminated with conoidin A-treated (or untreated) spermatozoa at 1 × 10^6^/mL, and incubated at 37 °C in 5% CO_2_ for 6 h to induce fertilisation. Following fertilisation, embryos were washed and incubated in 50 μL BM supplemented with 0.4% BSA in 5% CO_2_. The fertilisation rate was calculated as the number of zygote (single cell) to the number of inseminated oocytes. Finally, 18 h post-insemination, cleavage rate was calculated as the number of two-cell embryos to the number of zygote. Two-cell embryos were transferred to 50 μL BM supplemented with 0.4% BSA in 5% CO_2_. Five days after insemination, blastocyst development was estimated for each experimental group. The blastocyst formation rate was calculated as the number of blastocysts to the zygotes.

### Bioinformatics analysis

We used the Pathway Studio program (Elsevier, Amsterdam, The Netherlands) to identify protein-protein interactions, cellular regulation, and diseases associated with PRDXs. After inserting PRDXs as an input object, we searched for molecular cell interactions of PRDXs. Retrieved information was re-confirmed by checking every node-associated PubMed Medline hyperlink.

### Statistical analysis

Data were analysed using one-way ANOVA with SPSS statistical software (Version 12.0; Chicago, IL, USA). One-way ANOVA was used to analyse significant differences between the means of control and treatment groups. Tukey’s test was used to classify differences between groups as a multiple comparison. Differences between control and treated samples were considered significant at *P* values less than 0.05. Data are presented as mean ± SEM.

### Data availability

No datasets were generated or analysed during the current study.

## Electronic supplementary material

Supplementary Information
