# Supplementary material for: Peroxiredoxin activity is a major landmark of male fertility
Source: Sci Rep. 2017 Dec 7;7:17174. doi: 10.1038/s41598-017-17488-7 (PMC5719347; doi:10.1038/s41598-017-17488-7)

Supplemental Materials

Peroxiredoxin activity is a major landmark of male fertility

Do-Yeal Ryu, Ki-Uk Kim, Woo-Sung Kwon, Md Saidur Rahman, Amena Khatun, and Myung-Geol Pang*

Department of Animal Science & Technology, Chung-Ang University, Anseong, Gyeonggi-Do 456-756, Korea

*Correspondence *E-mail*: mgpang@cau.ac.kr; Tel: +82.31.670.4841; Fax: +82.31.675.9001

Fig. S1. Uncropped images of western blotting.

Figure 1C

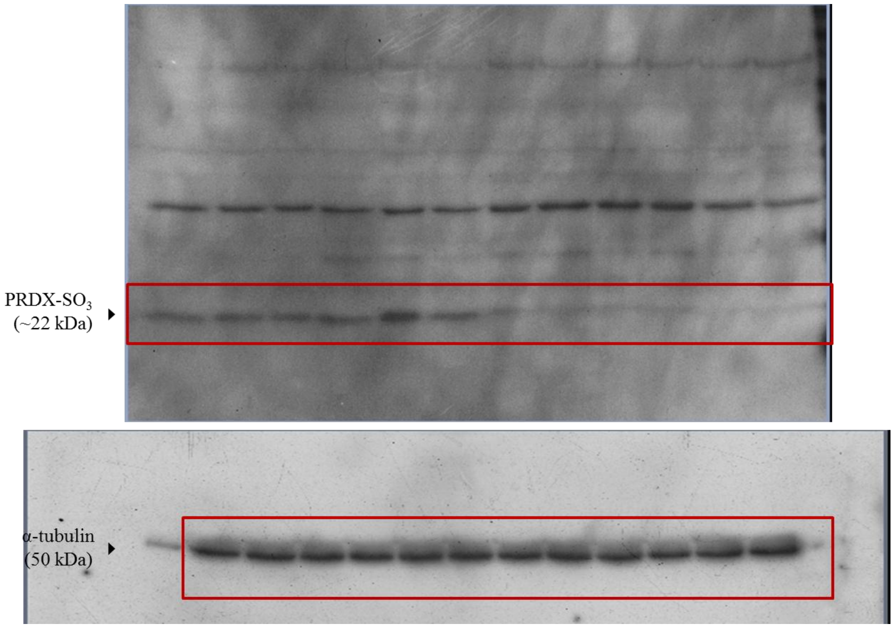

Figure 1D

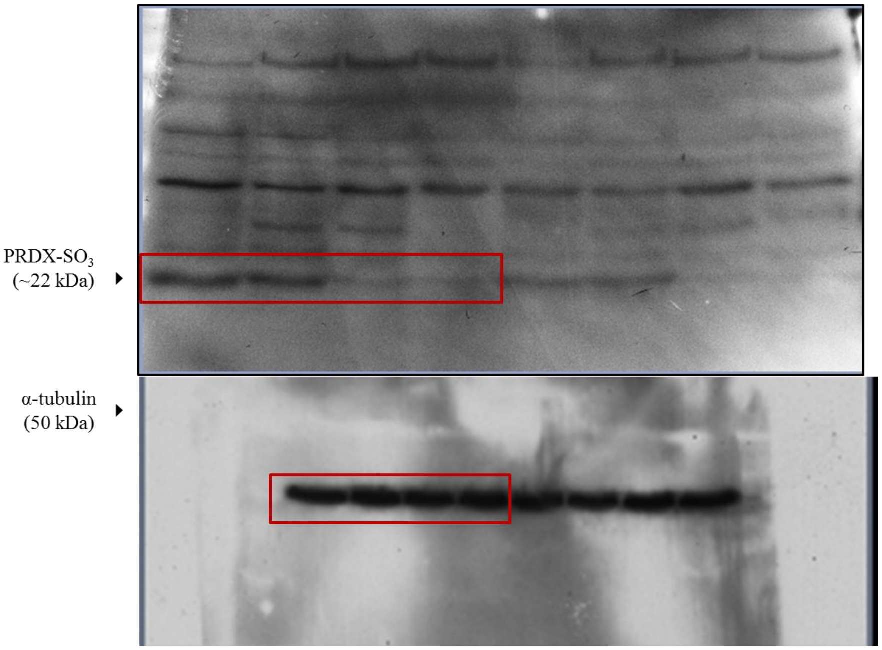

Figure 6B

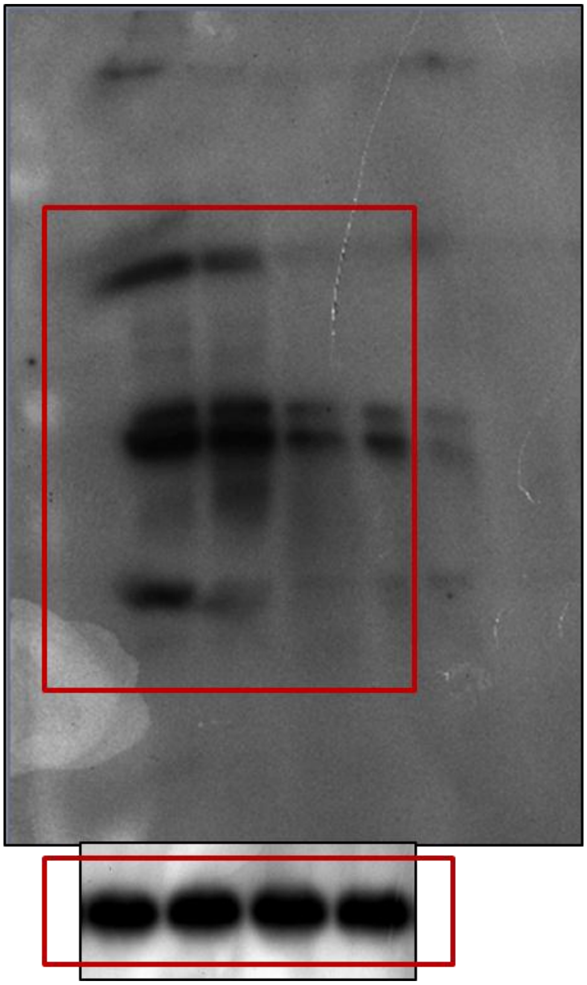

Figure 6D

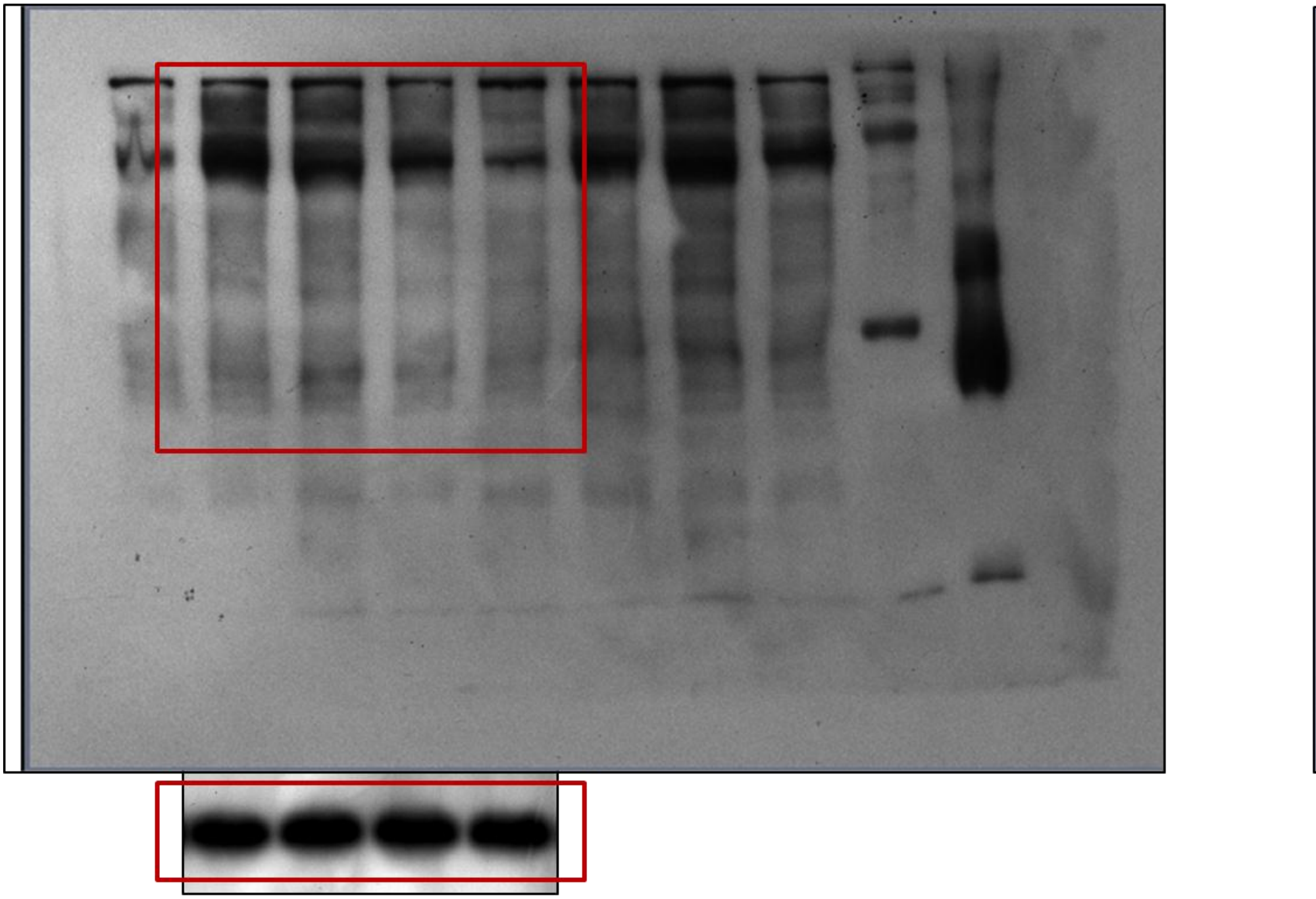

Supplement: Supplementary file 1 — Supplementary Information [file 41598_2017_17488_MOESM1_ESM.pdf]
